# Knowledge, and attitude as determinants of healthcare professionals’ self-medication practice to antibacterials in Tertiary Care hospitals, North West Ethiopia

**DOI:** 10.1038/s41598-025-88979-1

**Published:** 2025-02-12

**Authors:** Desalegn Getnet Demsie, Zenaw Debasu Addisu, Bereket Bahiru Tefera, Desye Gebrie, Etsay Weldekidan Tsegay, Adane Yehualaw, Kebede Feyisa, Malede Berihun Yismaw, Selamawit Yimer Kebede, Gizachew Motbaynor, Yazachew Engida, Abere Tilahun, Niguse Meles Alema, Getahun Mihret, Daniel Getasew, Nardos Bishaw, Chernet Tafere

**Affiliations:** 1 Department of Pharmacy, College of Medicine and Health Sciences, Bahir Dar University, Bahir Dar, Ethiopia; 2 Department of Pharmacy, College of Medicine and Health Sciences, Woldia University, Weldiya, Ethiopia; 3 Tigray Regional Health Bureau, Tigray, Ethiopia; 4 Department of Pharmacy, College of Medicine and Health Sciences, Adigrat University, Adigart, Ethiopia; 5 Department of Anesthesia, College of Medicine and Health Sciences, Adigrat University, Adigrat, Ethiopia; 6 St. Peter Hospital, Addis Ababa, Ethiopia

**Keywords:** Self-medication practice, Healthcare professionals, Antibiotics, Knowledge, Attitude, Health services, Patient education, Public health

## Abstract

The rise of antimicrobial resistance, driven largely by the inappropriate use of antibiotics, presents a significant global health challenge. Healthcare professionals (HCPs) self-medication practice (SMP) with antibiotics is a concerning practice. The role of knowledge, and attitudes, in shaping SMP has not been explored, in the context of Ethiopia. This study aims to investigate the patterns of antibiotic use, knowledge, attitudes, and associated the factors with SMP among healthcare professionals in tertiary hospitals in Bahir Dar, North West Ethiopia. A cross-sectional study was conducted from September 2023 to February 2024 in two tertiary hospitals in Bahir Dar, Ethiopia. The study included 410 healthcare professionals selected using proportional allocation and convenience sampling. A structured, self-administered questionnaire was used to assess participants’ demographics, knowledge, attitudes, and practices regarding antibiotic use. Knowledge was assessed through scoring, and attitudes were evaluated using a Likert scale. Data were analyzed using SPSS version 27.0, employing bivariate and multivariate logistic regression analyses to identify factors associated with SMA. Knowledge assessment revealed that 58.5% had good knowledge. In terms of job categories, nurses comprised the largest group (48.8%). A majority (60.2%) had 1–5 years of experience. 57.8% of participants exhibited a poor attitude to SMP. Respiratory infections (20.61%) were the most common health condition reported, followed by gastrointestinal infections (15.43%). The most frequently used antibiotics were amoxicillin (35%), augmentin (25%), and azithromycin (25%). Key factors influencing SMA included ease of access to antibiotics (36%), cost-effectiveness (23%), and knowledge/expertise (22%). Time constraints, perceived severity of conditions, and past self-medication experiences were also significant factors. While 83.8% considered self-medication to be safe, 75% recognized the potential adverse effects of medications. The multivariate analysis revealed that being a physician (AOR = 23.39) or a pharmacist (AOR = 7.79) was strongly associated with self-medication. Degree holders, MSc holders, and specialized physicians were also more likely to self-medicate. A poor attitude was a significant determinant, with healthcare professionals displaying poor attitudes being almost twice as likely to self-medicate (AOR = 1.91). The findings highlight the prevalent practice of self-medication with antibiotics among healthcare professionals in Ethiopia, influenced by factors such as knowledge, access to antibiotics, and professional attitudes. The study highlights the urgent need for targeted interventions to enhance healthcare professionals’ knowledge and attitudes regarding responsible antibiotic use while addressing their own practices of self-medication.

## Introduction

Antibacterials are among the most widely used drugs globally, significantly contributing to the reduction of mortality and morbidity^1^. However, the emergence of antimicrobial resistance (AMR) undermines these benefits, posing a major global public health threat. A significant proportion of people in developing nations are colonized with multidrug-resistant bacteria, such as *Escherichia coli*, *Salmonella spp.*, and *Streptococcus pneumoniae*. The problem of AMR is not confined to developing countries; the spread of resistant bacterial genes can also affect developed nations, further emphasizing the global nature of this issue^1^. A global report identifies AMR as a public health emergency, particularly in low- and middle-income countries, where it leads to higher mortality rates, prolonged hospital stays, and economic strain. In 2019, bacterial AMR contributed to an estimated 4.95 million deaths, with 1.27 million directly attributable to resistance^2^. By 2021, this figure rose to 4.71 million associated deaths, including 1.14 million directly due to bacterial resistance^3^. Furthermore, the World Bank estimates that, in a worst-case scenario, AMR could result in an additional one trillion US dollars in healthcare costs annually by 2050, highlighting the urgent need for concerted efforts to combat this pressing public health challenge^4^.

The misuse of antibacterials, particularly common in low- and middle-income countries, is a key driver of AMR. Reports reveal a growing trend in self-medication practice (SMP) with antibiotics^2,5–11^, which refers to the use of medications, including antimicrobials, for self-diagnosed conditions or the intermittent or continuous use of prescribed drugs for chronic or recurrent diseases^11,12^. Its prevalence varies between 10.25% and 100% among healthcare workers^1,13–15^, indicating that health professionals, including nurses, pharmacists, physicians, and laboratory technologists, engage in SMP. Such misuse carries significant risks, leading to severe adverse effects and serious health complications that can even be life-threatening. Additionally, SMP undermines the effectiveness of healthcare services and exacerbates the challenges posed by AMR^6,7^.

Previous studies have identified various factors contributing to SMP. Key risk factors include young age, gender differences^13^, and having relatives with medical backgrounds^11^. Financial constraints, a positive self-care attitude, and higher medical literacy can also motivate professionals to self-medicate instead of seeking formal treatment^6,12,14^. Additionally,, the perceived seriousness of an illness may encourage self-medication, particularly when individuals believe their condition is manageable^13^. Other influencing factors in low- and middle-income countries include the level of education, and monthly income, as well as issues related to accessibility and affordability of healthcare services^8,16^.

Despite the availability of numerous studies examining the prevalence of self-medication among healthcare professionals^13–15,17,18^, the specific prevalence of antibiotic self-medication and the role of knowledge, attitudes, and other factors in shaping SMP remain underexplored. This study is the first of its kind to investigate knowledge and attitude as a determinant of SMP with antibiotics specifically in Ethiopia, shedding light on the behavioral drivers of antibiotic misuse within the country’s healthcare system. By addressing this gap, the study provides critical insights into the factors influencing SMP and lays the groundwork for targeted interventions to combat AMR. Focusing on healthcare professionals is essential, as equipping them with the necessary knowledge and practices is a crucial first step toward educating the general public about the risks of self-medication and its impact on AMR.

Thus, this study aims to investigate the patterns of antibiotic use, as well as knowledge and attitudes as determinants of SMP among healthcare professionals in Tertiary Care Hospitals in Northwest Ethiopia.

## Methods

### Study design, study area and period

A facility-based cross-sectional study design was employed for this research. The study was conducted at Tibebe Ghion Comprehensive Specialized Hospital (TGCSH) and Felege Hiwot Specialized Hospital (FHSH) in Bahir Dar City, Amhara Region, Ethiopia, from September 30, 2023, to February 30, 2024. Bahir Dar, the capital of the Amhara Regional State, is located in the northwest of Ethiopia, approximately 560 km from Addis Ababa, the nation’s capital. The city spans a total area of 120 km², sits at an elevation of 1,800 m above sea level, and is geographically positioned at a latitude of 11.5742° N and a longitude of 37.3614° E. The total population of Bahir Dar is 290,437, comprising 142,068 men and 148,369 women. The city administration is organized into nine sub-cities and has a healthcare infrastructure that includes 10 public health centers, 2 public hospitals, and 2 private health institutions, providing essential health services to the residents of the region.

### Source and study population

The source population for this study comprised all healthcare in Tertiary Care Hospitals workers in Bahir Dar City. The study population included staff pharmacists, physicians, nurses, laboratory technologists, and midwives working across various units within two Tertiary Care Hospitals in Bahir Dar City during the study period. All healthcare workers, regardless of their years of work experience, were eligible to participate. However, those on sick leave, absent for other reasons during the data collection period, or unwilling to participate were excluded. A six-month recall period was applied to gather information from all participants, ensuring the reliability of self-reported behaviors and practices.

### **Sample size determination**, sampling procedure

The sample size was determined using the single population proportion formula. Assuming a proportion (p) of 50% to maximize variability, a 95% confidence level (Z = 1.96), a margin of error of 0.05, and accounting for a 10% nonresponse rate, the final calculated sample size was 410.

The study population consisted of 1,326 healthcare workers across the two hospitals, distributed as follows: 298 physicians, 126 pharmacists, 646 nurses, 74 laboratory technologists, and 181 midwives. Proportional allocation was applied to ensure representativeness of the sample, resulting in the following breakdown: 92 physicians, 39 pharmacists, 200 nurses, 23 laboratory technologists, and 56 midwives, totaling 410 participants.

The total numbers of each professional group were based on official records from the two Tertiary Care Hospitals. Participants were selected using a convenience sampling method, which involved recruiting individuals who were readily accessible and willing to participate during the study period.

### Validity and reliability

The questionnaire was developed by DGD and CT after an extensive review of literature related to similar studies. A panel consisting of two clinical pharmacy lecturers and a physician critically appraised the data collection tool, evaluating its relevance, accuracy, and appropriateness. Face validity was established by presenting the questionnaire to 20 health workers from the departments of medicine, pharmacy, nursing, midwifery, and medical laboratory technology. Responses from the pretest were excluded from the final analysis.

The internal consistency of the questionnaire was assessed using Cronbach’s alpha, which yielded a satisfactory reliability score (α = 0.75). The questionnaire was structured into seven sections. The first section collected demographic data, including job category, age, gender, and education level. Sections two, three, and four focused on assessing the indications for antibiotic use, types of antibiotics used, and influencing factors, respectively. The sixth section examined healthcare workers’ attitudes toward self-medication, while the seventh evaluated participants’ knowledge of self-medication practices.

Knowledge responses were scored on a scale ranging from 0 to 7. Participants answered knowledge-related items with “Yes,” “No,” or “Do not know.” A score of 1 was assigned for “Yes,” while “No” and “Do not know” were scored as 0. Knowledge levels were categorized as poor for scores of 0–4 and good for scores of 5–7. Attitudes were assessed using a five-point Likert scale, with responses scored as follows: 5 for “Strongly agree,” 4 for “Agree,” 3 for “Uncertain,” 2 for “Disagree,” and 1 for “Strongly disagree. The mean score was used as a benchmark to categorize attitudes as either poor or good. Participants whose scores fell below the mean were classified as having poor attitudes.

### Study variables

The dependent variable in this study was the prevalence of self-medication. Independent variables included a range of factors: healthcare workers’ attitudes and knowledge, socio-demographic characteristics (such as age, sex, religion, level of education, income, job category, and work experience), as well as specific parameters related to their attitudes and knowledge on self-medication.

### Data collection tools and data collection procedure

Data were collected from the study participants using a pre-tested, structured, self-administered questionnaire adapted from previous research on similar topics^3,5,15,19^. The data collection tools were distributed to the participants and subsequently collected by the principal investigator. Verbal consent was obtained from all participants.

### Data processing and analysis

Data entry and cleaning were performed using EpiData version 3.1. Before data entry, the data were thoroughly checked for completeness, and any inconsistencies or missing values were addressed. Incomplete questionnaires were excluded from the analysis.

Data analysis was carried out using SPSS version 27.0. Bivariate logistic regression was used to examine the associations between each independent variable and the dependent variable. In the univariate analysis, each factor was assessed for its potential association with the outcome variable, with a p-value of less than 0.2 used as a threshold to select predictors for inclusion in the multivariate analysis. The crude odds ratio (COR) and adjusted odds ratio (AOR) were calculated, and a p-value of less than 0.05 was considered statistically significant.

## Result

The majority of participants were aged 24 to 30 years (64.6%). Regarding education, 67.1% had a degree, and 6.3% held a diploma. Knowledge levels indicated that 58.5% had good knowledge. Nurses accounted for 48.8% of the participants, while doctors made up 22.4%. In terms of attitude, 57.8% exhibited poor attitude (Table 1).

**Table 1:** Sociodemographic characteristics of healthcare professionals at TGCSH and FHSH (*N* = 410).

Variable		Frequency (%)	Variable		Frequency (%)
Age	24–30	265 (64.6)	Job category	Doctor	92 (22.4)
	31–40	137 (33.4)		Pharmacist	39 (9.5)
	> 41	8 (2)		Nurse	200 (48.8)
Sex	Male	204 (49.8)		Midwife	56 (13.7)
	Female	206 (50.2)		Laboratory technologist	23 (5.6)
Education	Diploma	26 (6.3)	Experience	< 1 year	7 (1.7)
	Degree	275 (67.1)		1–5 year	247 (60.2)
	Masters	76 (18.5)		5–10 year	129931.5)
	Specialized	33 (8)		> 10 year	27 (6.6)
Knowledge	Poor	170 (41.5)	Attitude	Poor	237 (57.8)
	Good	240 (58.5)		Good	173 (42.2)

Respiratory infections affected 20.61% of the individuals followed by gastrointestinal infections (15.43%). A smaller proportion of the participants experienced nausea and vomiting (2.10%), aches and pains (3.84%), ear infections (5.57%), and typhoid and typhus (2.00%) (Table 2).

**Table 2:** Indications for self-medication practices among healthcare professionals at TGCSH and FHSH (*N* = 410).

Condition	%	Condition	%
Respiratory infections	20.61	Wound infections	6.19
UTI	7.43	Unidentified conditions	2.08
Gastrointestinal infections	15.43	Common cold	8.07
Skin infections	9.65	Aches and pains	3.84
Dental infections	6.19	Tonsilitis	7.42
STIs	4.33	Nausea and vomiting	2.10
Ear infections	5.57	Sore throat and tonsillitis	12.66
Fever of unknown origin	9.90	Typhoid and typhus	2.00
		Wound infections	6.19

STI, sexually transmitted infections; UTI, urinary tract infection.

**Fig. 1:**
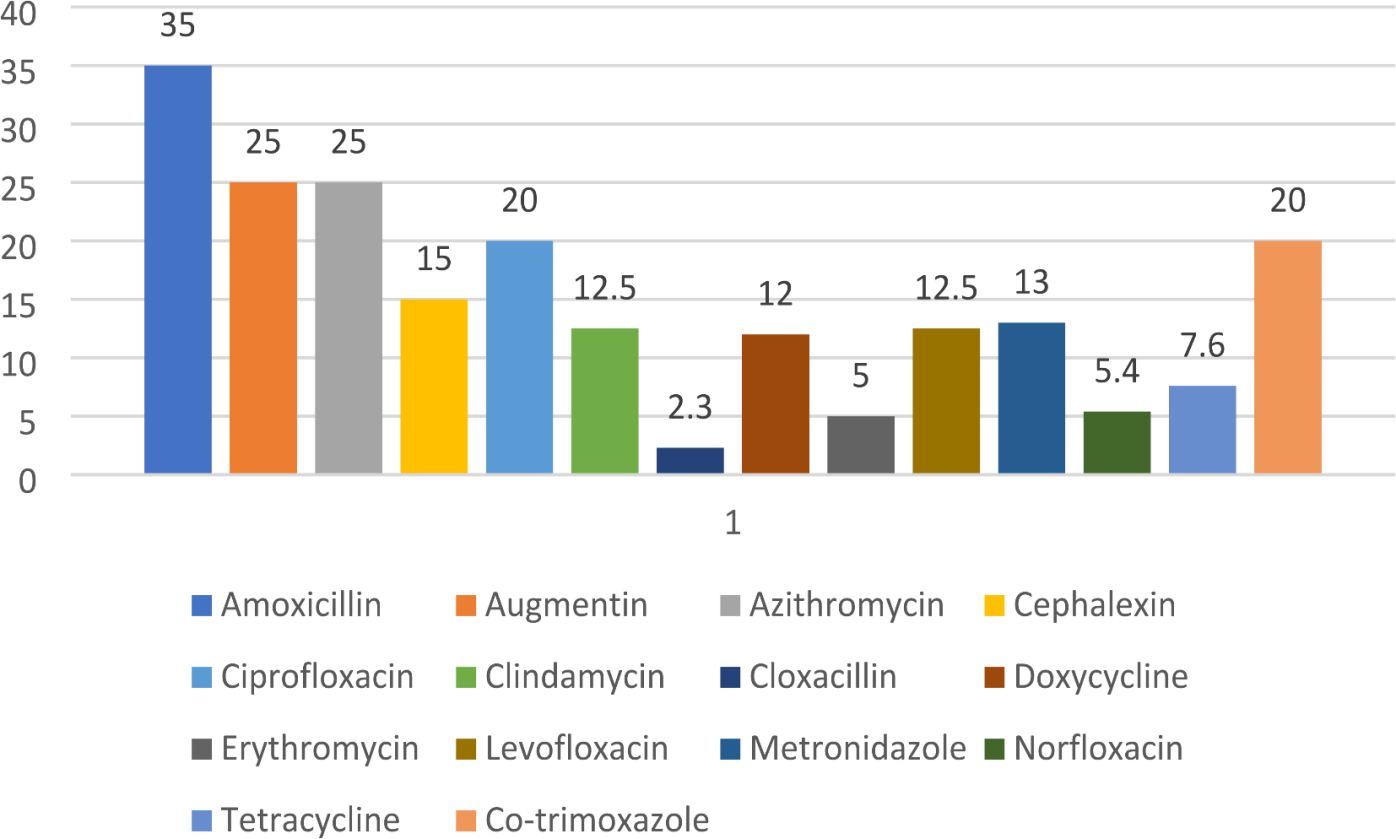
Pattern of antibiotic use at TGCSH and FHSH.

As indicated in Fig. 1 amoxicillin was the most commonly used antibiotic, accounting for 35% of the total usage. Both augmentin and azithromycin were equally prescribed, each constituting 25%. Norfloxacin and erythromycin were less frequently prescribed, at 5.4% and 5%, respectively. Cloxacillin was the least used, constituting only 2.3% of the total antibiotic usage.

**Fig. 2:**
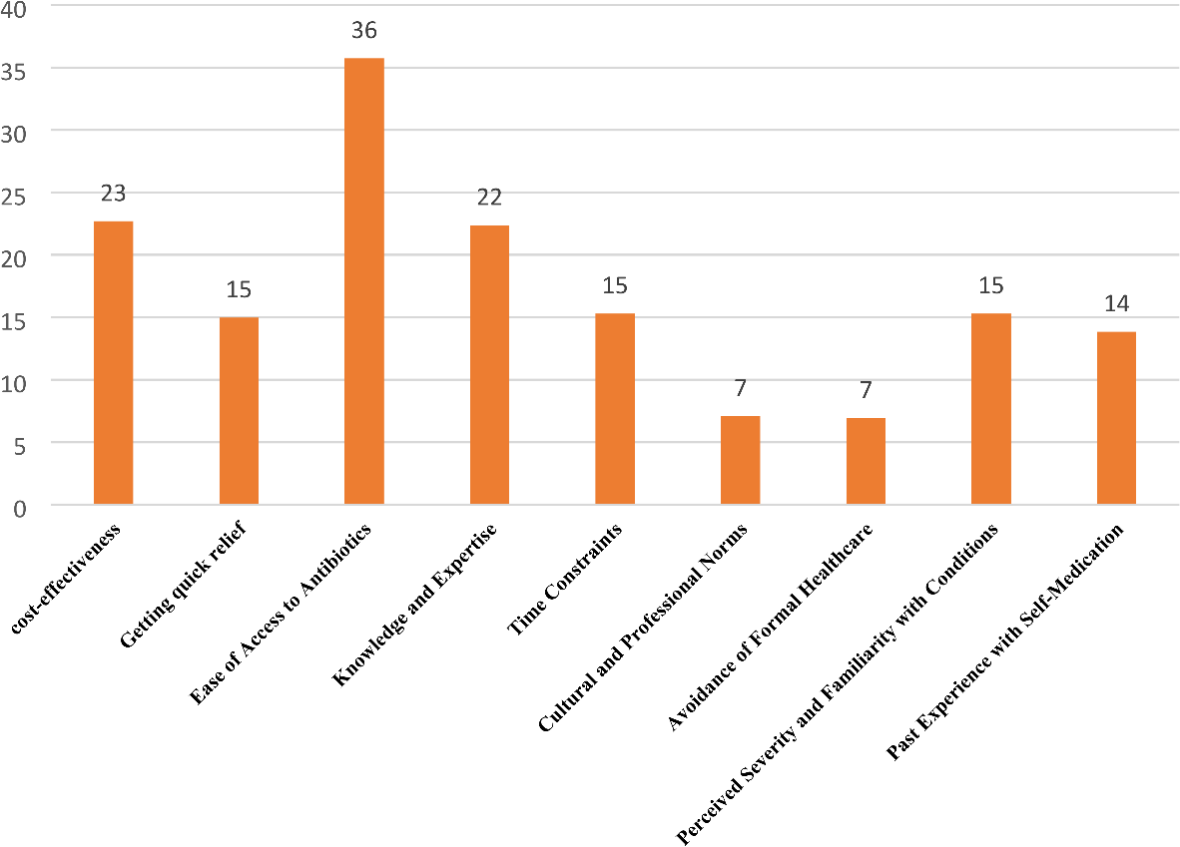
Influential factors for antibiotic self-medication.

Figure 2 revealed that the most significant factor contributing to antibacterial self-medication among healthcare professionals was the ease of access to antibiotics (36%). Cost-effectiveness was the second most influential factor (23%). Knowledge and expertise also played a considerable role, influencing 22% of healthcare professionals.

Cultural and professional norms and the avoidance of formal healthcare were less frequently mentioned, each contributing 7% to the practice of self-medication. The mean score of healthcare professionals was 2.94 out of 5, revealing that 237 professionals exhibited a poor attitude toward self-medication (Fig. 2).

**Fig. 3:**
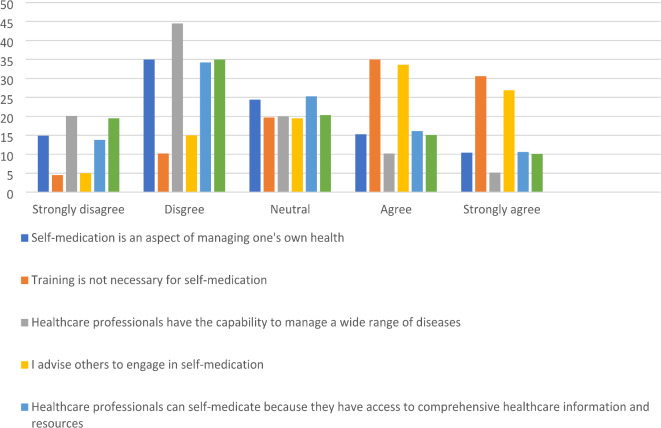
Healthcare workers’ attitudes towards self-medication (*N* = 410).

Figure 3 showed that 14.9% of participants strongly disagree with notion that that self-medication as an integral part of managing one’s own health and 35.0% disagree with this perspective. However, there is a notable diversity in opinions, as 24.4% remained neutral, and 25.7% agreed or strongly agreed. Regarding the necessity of training for self-medication, responses were divided. Only 4.5% strongly disagreed that training is unnecessary, while 10.2% disagreed with this view. In contrast, 35.0% agreed, and 30.6% strongly agreed.

Most healthcare professionals’ express confidence in their ability to manage a wide range of diseases, with 20.1% strongly disagreeing and 44.5% disagreeing with this statement. This confidence is reflected in their practices, as 64.6% of respondents believe they have the capability to handle various health issues. When it comes to advising others to self-medicate, only 5.0% strongly disagree and 15.0% agree with recommending this practice. A larger proportion, 60.5%, agrees or strongly agrees with endorsing self-medication for others, suggesting caution in promoting this approach.

Access to comprehensive healthcare information and resources is seen as a justification for self-medication by 13.8% of respondents who strongly agree and 34.2% who agree. However, 26.7% disagree or strongly disagree, reflecting some concern about the appropriateness of self-medication even with access to resources. Approximately 19.5% strongly agree and 35.0% agree that availability of over-the-counter (OTC) medicines and the confidence in their safety encourage their self-medication, while 25.2% are neutral or disagree with this statement (Fig. 3).

**Fig. 4:**
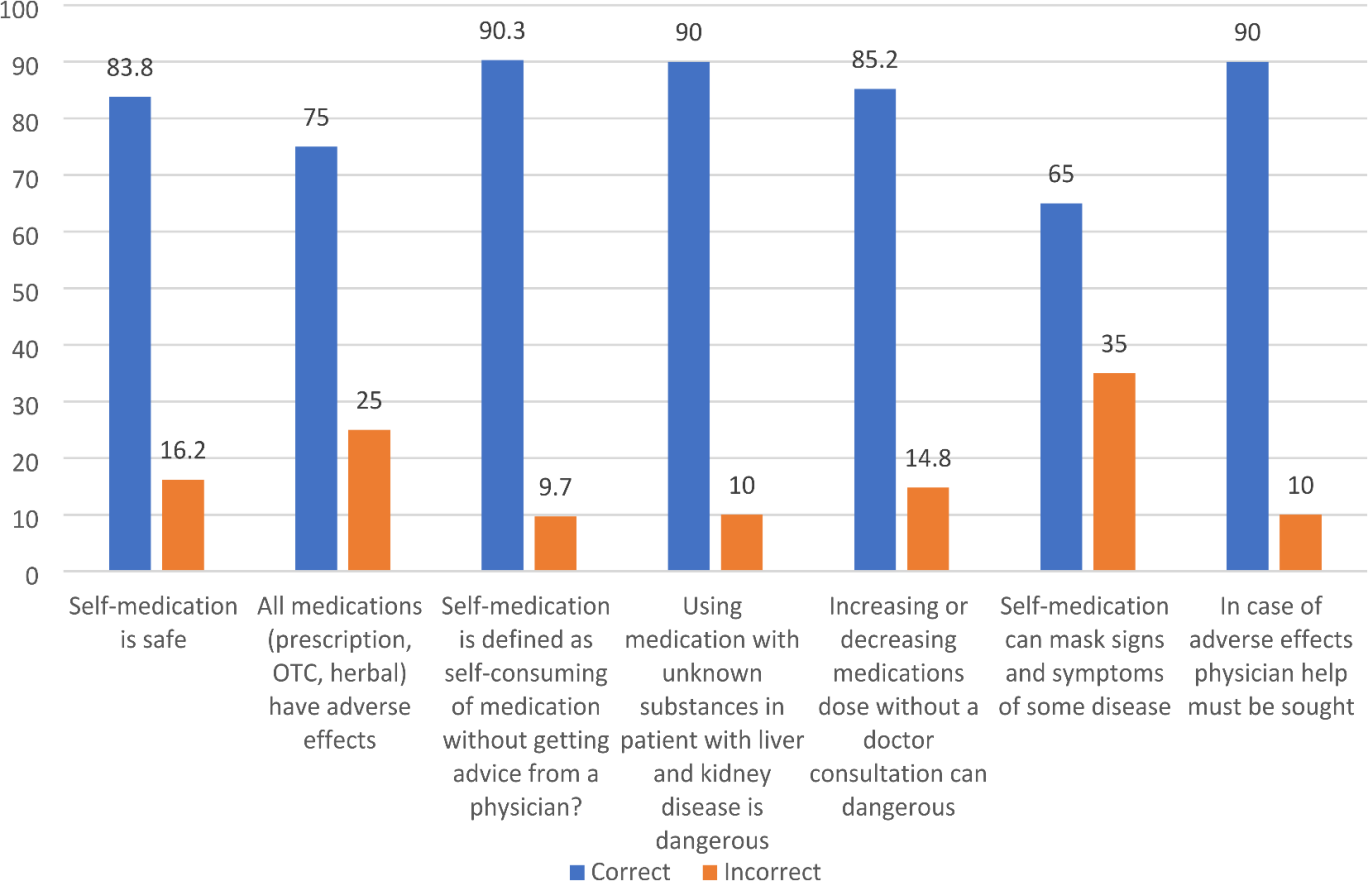
Participants’ knowledge related to self-medication. *N* = 410.

The majority of respondents, 83.8%, consider self-medication to be safe (Fig. 4). Regarding the awareness of adverse effects, 75% of healthcare professionals recognize that all types of medications can have adverse effects. 90.3% of respondents correctly identified self-medication as the use of medication without consulting a physician.

The potential dangers of using medications with unknown substances in patients with liver and kidney diseases are well recognized by 90% of respondents. Additionally, 85.2% of healthcare professionals understand that altering medication doses without a doctor’s consultation can be dangerous. However, there is less consensus on the impact of self-medication on disease symptom recognition. While 65% of respondents acknowledge that self-medication can mask signs and symptoms of certain diseases, 35% do not recognize this risk (Fig. 4).

**Table 3:** Antibiotics self-medication and associated factors among healthcare professionals in Tertiary Care hospitals, Northwest Ethiopia (*N* = 410).

		Self-medication		Total	P-v	COR	CI		P-v	AOR	CI	
		No	Yes				Lower	Upper			Lower	Upper
Job category	Doctor	19	73	92	0.000	8.78	3.16	24.39	0.000	23.393	6.626	82.587
	Pharmacist	9	30	39	0.001	7.61	2.39	24.28	0.001	7.785	2.301	26.343
	Nurse	90	110	200	0.031	2.79	1.101	7.087	0.044	2.809	1.029	7.669
	Midwife	40	16	56	0.868	0.914	0.317	2.641	0.785	0.854	0.276	2.647
	Lab	16	7	23								
Age	24–30	109	156	265	0.078	4.29	0.851	21.672	0.002	23.87	3.214	177.29
	31–40	59	78	137	0.099	3.96	0.773	20.357	0.003	20.34	2.753	150.36
	> 41	6	2	8								
Education status	Diploma	21	5	26								
	Degree	120	155	275	0.001	5.42	1.988	14.80	0.000	21.03	5.752	76.940
	Masters	23	53	76	0.000	9.67	3.250	28.82	0.000	40.392	10.084	161.79
	Specialized	10	23	33	0.000	9.66	2.836	32.90	0.000	20.62	4.697	90.53
Knowledge	Poor	55	115	170	0.001	2.05	1.366	3.09	0.414	1.268	0.718	2.239
	Good	119	121	240								
Attitude	Poor	82	155	237	0.000	2.14	1.438	3.206	0.023	1.909	1.094	3.330
	Good	92	81	173								

As shown in the Table 3, being a physician (AOR = 23.393; 95% CI: 6.626, 82.587) or a pharmacist (AOR = 7.785; 95% CI: 2.301, 26.343) was strongly associated with self-medication practices. Specifically, physicians were 23 times more likely to self-medicate compared to medical laboratory technologists. Additionally, a significant association was observed among degree holders (AOR = 21.03; *p* < 0.001), MSc holders (AOR = 40.392; *p* < 0.001), and physicians with a specialty (AOR = 20.62; *p* < 0.001). Although poor knowledge was significantly associated with self-medication in univariate analysis, this association did not remain significant in the multivariate analysis. However, a poor attitude was a significant determinant of self-medication practices (COR = 2.14; 95% CI: 1.438, 3.206; AOR = 1.909; 95% CI: 1.094, 3.330) (Table 3).

## Discussion

Self-medication is considered an aspect of self-care, as acknowledged by the World Health Organization^17,20^, particularly in environments where healthcare personnel are limited. In such cases, allowing patients to manage minor health issues on their own can alleviate the strain on healthcare systems, reducing both costs and wait times for care. However, self-medication carries significant risks, including adverse drug reactions, drug interactions, incorrect dosing, polypharmacy, and improper or excessive use of medications^17,21,22^. One of the major concerns is the improper use of antibiotics, which requires precise diagnosis and appropriate prescription to avoid contributing to antimicrobial resistance. Misuse of antibiotics can lead to the loss of affordable and effective treatments, driving the demand for more expensive alternatives^23^.

In our study 236 out of 410 health workers self-medicate with antibacterials (57.56%). The finding was lower than SMP among HCPs in wester Ethiopia (73.4%)^13^, Debre Markos(72.2%)^18^, and Nekemte reported lower(67.5%)^24^. These higher prevalence rates might be due to differences in income, where 32.5% of respondents had financial constraints^24^, compared to 23% of respondents with cost-effectiveness concerns in our finding. Additionally, perceived severity of ill ness in our case is 15% compared to SMP practice in west Ethiopia Hospitals (40.7%)^13^ may cause greater proportions of SMP. Furthermore, disparities in disease pattern, as these findings assessed the SMP among HCP in all medication types, my result in differences in the magnitude of SMP^1,2,25,26^.

Overall, 58.5% were aware of what self-medication was which is lower than a finding by Mustafa et al., 2023(86.4%)^15^ and 42.2% had good attitude towards antibiotic SMP. Knowledge and attitude of health workers affects the magnitude of self-medication to antimicrobial medications. In our study, the rates of poor knowledge and attitude were higher among healthcare professionals at 42.5% and 57.8%, respectively. Poor knowledge was significantly associated in univariate analysis, and poor attitude was a significant determinant of self-medication practices (AOR = 1.909; 95% CI: 1.094, 3.330). The higher record of poor knowledge in our findings is probably stemmed from respondents’ significantly higher proportion responded that self-medication cannot mask the signs and symptoms of some diseases (35%). The other 25% know that over-the-counter medication do not have adverse effects, while 16% of the participants from are aware of that self-medication to antibacterials is safe. Additionally, 14% are not the risk of increasing or decreasing doses without prescribers’ consultation, and 9.7% did not even properly define self-medication.

Our study highlighted the influence of healthcare professionals’ attitudes on self-medication practices. A significant portion, 35%, believe that self-medication is an acceptable part of managing one’s own health, with 14.9% expressing strong disagreement with this perspective. This attitude may stem from a sense of professional capability, as 44.5% of respondents disagreed and 20.1% strongly disagreed with the notion that healthcare professionals can manage a broad spectrum of illnesses independently. Despite the risks associated with self-medicating antimicrobials, a notable number of professionals do not perceive additional training on this issue as necessary; 35% agreed, and 30.6% strongly agreed with the need for such training. Additionally, access to comprehensive healthcare information and resources was viewed as a justification for self-medication by some professionals, with 34.2% disagreeing and 13.8% strongly disagreeing. Lastly, the easy availability of medications was another contributing factor, with 34.2% disagreeing and 19.8% strongly disagreeing that this accessibility encourages self-medication practices.

In our study, we observed that self-medication with antibiotics among healthcare professionals is notably influenced by several key factors. The most prominent driver, cited by 36% of respondents, was the ease of access to antibiotics. This suggests that the availability of these drugs within healthcare settings may contribute to increased self-prescription practices, as healthcare professionals often have direct access to these medications. Finds also support that the availability and uncontrolled access to all kinds of medicines especially prescription-only type exacerbates self-medication practice^5,17^.

Cost-effectiveness was identified as the second leading factor, influencing 23% of respondents. This indicates that healthcare professionals may choose self-medication as a more affordable alternative to seeking formal consultation and treatment^5,17,26^. Additionally, the familiarity and expertise of healthcare professionals regarding antibiotics, each reported by 22% of respondents, appear to play significant roles. Other study also reported that when symptoms of previous infection re-appear or a member of the family or a friend has the same symptoms, healthcare workers indulge in self-medication practices^16^. These factors reflect a confidence in their own knowledge and judgment, which may lead them to self-prescribe antibiotics without seeking additional medical input^5^.

Our study revealed strong associations between specific professional roles, educational backgrounds, and the likelihood of engaging in self-medication with antibiotics. Physicians were notably prone to self-prescription, being 23 times more likely to self-medicate than other healthcare professionals. This result is in agreement with a study in China, where professionals who perceived as having moderate to high antibiotic knowledge were more likely to self-medicate themselves^14^. This tendency could be attributed to their high level of medical expertise and the autonomy they experience, which may make them more comfortable self-managing minor health issues without seeking external advice. Pharmacists, likewise, showed a significantly higher likelihood of SMP, being nearly 8 times more likely than other healthcare professionals. This could be due to their direct access to medications and confidence in their pharmacological knowledge, leading them to view self-treatment as adequate. Of course, this habit of pharmacists strengthened self-medication practice by the general public^2,7,9,10,26,27^. As pharmacists attitude to self-medication prevail, their impact on filling non-prescription antibiotics increase, partly due to the high demand and high selling benefit from these medications^16^.

Educational attainment also played a critical role, as degree holders were 21 times, MSc holders 40 times, and physicians with a specialty 20 times more likely to engage in SMP compared to those with less education. This trend suggests that advanced qualifications may foster a sense of self-reliance and confidence in healthcare professionals, potentially prompting them to bypass conventional consultations. Negligence to appropriate use of antibiotics by healthcare workers has burden to the antimicrobial stewardship program underway in many parts of Africa. As the professionals themselves tend to practice self-medication, their efforts to teach patients not self-medicate will most likely be lower. This adds up to the problem of lower doctors to patients ratio in low-income settings[15], which leads to the number of patients who have access to doctors are few and it further implies that the few doctors will be stretched and patient will most likely resort to self-medication^5,10,17^.

## Conclusion and recommendation

This study highlights the widespread practice of self-medication with antibiotics among healthcare professionals in tertiary hospitals in Bahir Dar, Ethiopia. Despite a significant portion of participants demonstrating good knowledge, a notable number exhibited poor attitudes towards self-medication, which was identified as a key predictor of this behavior. Factors such as easy access to antibiotics, time constraints, and the perceived effectiveness of self-medication contributed to this practice, with physicians and pharmacists being particularly likely to self-medicate. The findings underscore the urgent need for targeted interventions to enhance healthcare professionals’ knowledge and attitudes toward responsible antibiotic use. It is essential to implement educational programs that emphasize the risks of antimicrobial resistance and promote better attitudes and practices regarding antibiotic use. Hospitals should also adopt policies to restrict access to antibiotics without proper prescriptions and ensure adherence to antibiotic stewardship guidelines. Furthermore, ongoing research is necessary to investigate the underlying psychological and systemic factors that contribute to self-medication, while continuous monitoring of healthcare professionals’ practices should be conducted to refine interventions and policies.

## Strength and limitations

This study provides valuable insights into the prevalence and factors influencing SMP among healthcare professionals in Ethiopia, a topic that has been underexplored in this context. It is the first to examine the role of knowledge, and attitudes in shaping SMP, offering a detailed understanding of how these factors contribute to antibiotic misuse. The inclusion of diverse healthcare professionals from multiple disciplines (doctors, pharmacists, nurses, laboratory technologists, and midwives) strengthens the generalizability of the findings. Additionally, the use of multivariate analysis allows for a more nuanced understanding of the factors associated with SMP, helping to identify key areas for targeted interventions. However, the cross-sectional design limits the ability to establish causality, as it captures data at a single point in time. The use of self-reported questionnaires may introduce response bias, as participants might underreport or overreport their behaviors. Furthermore, the convenience sampling method may limit the representativeness of the sample, potentially affecting the generalizability of the findings to other regions or settings in Ethiopia. Despite these limitations, the study sheds light on important behavioral drivers of antibiotic misuse, and further research with longitudinal designs and more diverse sampling methods is needed to confirm the results and explore the long-term effects of interventions.

## Data Availability

The datasets generated and analyzed during this study are accessible and can be obtained from the corresponding author upon request.
